# Using the Kriging Correlation for unsupervised feature selection problems

**DOI:** 10.1038/s41598-022-15529-4

**Published:** 2022-07-07

**Authors:** Cheng-Han Chua, Meihui Guo, Shih-Feng Huang

**Affiliations:** 1 Department of Applied Mathematics, National Sun Yat-sen University, Kaohsiung, 80424 Taiwan, ROC; 2 Department of Applied Mathematics, National University of Kaohsiung, Kaohsiung, 811 Taiwan, ROC

**Keywords:** Classification and taxonomy, Machine learning, Statistical methods

## Abstract

This paper proposes a KC Score to measure feature importance in clustering analysis of high-dimensional data. The KC Score evaluates the contribution of features based on the correlation between the original features and the reconstructed features in the low dimensional latent space. A KC Score-based feature selection strategy is further developed for clustering analysis. We investigate the performance of the proposed strategy by conducting a study of four single-cell RNA sequencing (scRNA-seq) datasets. The results show that our strategy effectively selects important features for clustering. In particular, in three datasets, our proposed strategy selected less than 5% of the features and achieved the same or better clustering performance than when using all of the features.

## Introduction

Feature selection for unsupervised learning is a challenging problem. In this study, we propose a Kriging-Correlation (KC) Score, which integrates the Automatic Fixed Rank Kriging (AutoFRK)^1^ method with a correlation analysis, to measure feature importance in clustering analysis. A KC Score-based feature selection strategy is further developed for extracting important features for high dimensional clustering analysis. The feature selection procedure includes three main steps: calculating the importance score of each feature, ordering the features by their scores, and deciding the number of features to be selected. In addition, the proposed strategy also suggests an appropriate kernel to enhance the clustering accuracy and efficiency.

To investigate the performance of the proposed strategy, we study four single-cell RNA sequencing(scRNA-seq) datasets to extract the critical features for cell type clustering analysis. Cell type identification has many applications, including helping to understand how different cells function and interact. A classical and straightforward approach is to assign cells their types by micromanipulation, but this method is usually time-consuming and risks mislabeling. Recently, a data-driven approach, single-cell interpretation via multi-kernel learning (SIMLR)^2^, proposes to cluster cells based on single-cell RNA sequences and then to accordingly identify the cell type for each cluster. This approach not only saves time by not requiring cell-by-cell identification, but it is also able to identify some undiscovered cells associated with cancer^3^. For scRNA-seq data, there are very few samples *n* compared to the number of genes (features) *p*. A dimension reduction method is often employed to circumvent this low *n*/*p* ratio when conducting clustering analysis. The SIMLR study accomplished dimension reduction through the well-known t-distributed stochastic neighbor embedding (t-SNE)^4^ method. Since gene expression in scRNA-seq data usually contains dropout events (zero measurements), SIMLR adopts a multi-resolution Gaussian kernel to measure the similarity matrix used in the t-SNE.

After conducting clustering analysis in the latent space obtained by t-SNE with SIMLR, we propose a strategy to find important genes in cell clustering. The results show that our strategy effectively selects important features for clustering. In particular, in three datasets, less than 5% of features are selected by our proposed strategy, yet the classification accuracy and Normalized Mutual Information (NMI) is the same or better than using all features. Furthermore, for the four datasets, the KC Score has either comparable or better NMI than the Laplacian Score^5^, which is one of the well-known locality preserving filtering methods for unsupervised feature selection.

## Results

### Datasets

In this study, we apply the proposed method to four published scRNA-seq datasets, which record gene expression for different kinds of cell species. The numbers of subjects *n*, genes (or features) *p*, number of classes, and descriptions of the datasets are given in Table  1. From Table  1, one can see the aforementioned low *n*/*p* ratio in each dataset. The datasets analyzed in this study are available in the Supplementary information section on the website https://www.nature.com/articles/nmeth.4207.

**Table 1:** Description of four scRNA-seq datasets.

Data name	*n*	*p*	Class #	Description
mECS	182	8989	3	Embryonic stem cells under different cell cycle stages
Kolod	704	13,473	3	Pluripotent cells under different environment conditions
Pollen	249	6982	11	Eleven cell populations including neural cells and blood cells
Usoskin	622	17,772	4	Neuronal cells with sensory subtypes

### Features selection procedure

Let $Z^{{M}}(X)$ denote the projection of the original data *X* (see Eq. (1)) on the 2-dimensional latent space by t-SNE with the similarity matrix $S^{{M}}(X)$ obtained via SIMLR in Eq. (3), where the superscript *M* represents all results generated from SIMLR. Let ${\hat{y}}^{{M}}(X)$ be the clustering label vector of the cells by applying k-means on $Z^{{M}}(X)$. For each gene, we calculate its Laplacian Score by $S^{{M}}(X)$ and KC Score by $Z^{{M}}(X)$, details are given in “Methods” section.

Let ${\mathcal {V}}_k$ be the feature collection of the first *k* highest KC Scores; hence ${\mathcal {V}}_k$ is a submatrix of *X* with dimension $n \times k$. Let $S^{G}({\mathcal {V}}_k)$ be the similarity matrix estimated by the single Gaussian kernel method based on the ${\mathcal {V}}_k$, where the superscript *G* represents all results generated from a single Gaussian kernel method. Our goal is to find genes that play important roles in clustering by KC Scores. Details (Strategy A) are given below.

**[Strategy A]**

1. For each $k=1,\ldots ,p$, apply t-SNE on ${\mathcal {V}}_k$ with $S^{G}({\mathcal {V}}_k)$ and denote the associated latent space projection as $Z^G({\mathcal {V}}_k)$. Then apply k-means on $Z^G({\mathcal {V}}_k)$ to obtain the clustering label vector ${\hat{y}}^G({\mathcal {V}}_k)$.
2. For each $k=1,\ldots ,p$, calculate the Pillai’s trace in MANOVA for ${\hat{y}}^G({\mathcal {V}}_k)$ and $Z^G({\mathcal {V}}_k)$ and denote the result as ${\mathcal {F}}({\mathcal {V}}_k)$. Let $k_1=\mathrm{arg}\max _{k=1,\ldots ,p}{\mathcal {F}}({\mathcal {V}}_k)$.
3. To further prune ${\mathcal {V}}_{k_1}$, consider $k_2=1+\text {max}\left\{ k<k_1\ :\ \text {NMI}\left( {\hat{y}}^G({\mathcal {V}}_k),{\hat{y}}^G({\mathcal {V}}_{k_1})\right) < 0.95\right\}$.
4. If $\text {NMI}({\hat{y}}^G({\mathcal {V}}_{k_2}),{\hat{y}}^{{M}}(X))> 0.7$, then output ${\mathcal {V}}_{k_2}$ and ${\hat{y}}^G({\mathcal {V}}_{k_2})$; otherwise, go back to steps 1-3, replacing the superscript *G* by *M* and output ${\mathcal {V}}_{k_2}$ and ${\hat{y}}^{{M}}({\mathcal {V}}_{k_2})$.

**Figure 1:**
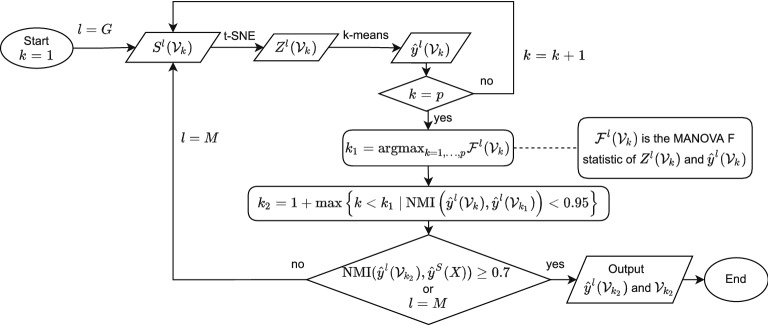
Flow chart of Strategy A.

Figure 1 presents the flow chart of the above procedure. In step 1, the reasons for using $S^{G}$ are twofold: $S^{G}$ requires lower computational costs than $S^{{M}}$; the clustering performances of $S^{G}$ and $S^{{M}}$ are comparable when the number of critical genes ($k_2$ in step 3) is small. In step 2, we decide the initial features set ${\mathcal {V}}_{k_1}$ by maximizing the Pillai’s trace statistics in MANOVA, which corresponds to the variance ratio of between-group and within-group. Since ${\mathcal {V}}_{k_1}$ still possibly contains some irrelevant or non-significant features, we adopted a pruning step in Strategy A. The pruning step is a widely used technique to further refine the selected features in model selection^6^ and tree-based methods in machine learning^7^. It aims to reduce variance and avoid overfitting by deleting some irrelevant or non-significant features. Therefore, we pruned the set ${\mathcal {V}}_{k_1}$ to ${\mathcal {V}}_{k_2}$ in step 3 of Strategy A such that after dropping unimportant genes in ${\mathcal {V}}_{k_1}$, the NMIs between ${\hat{y}}^G({\mathcal {V}}_{k_1})$ and ${\hat{y}}^G({\mathcal {V}}_{k})$ are greater than 0.95 for all $k\in [k_2,k_1]$. In step 4, we check the adequacy of ${\mathcal {V}}_{k_2}$ by comparing the NMI between ${\hat{y}}^G({\mathcal {V}}_{k_2})$ and ${\hat{y}}^{{M}}(X)$ to decide whether to replace superscript *G* by *M* in steps 1–3. Similarly, to find the set of critical genes, denoted by ${\mathcal {V}}_{k_2}^*$, in clustering by Laplacian Scores, we only need to replace the KC Scores by the Laplacian Scores in the above procedure.

### Performance of Strategy A

We mainly use two metrics, NNA^2^ (under supervised setting) and NMI^2^ (under unsupervised setting), to compare the classification and clustering performance based on the latent space projections $Z^l({\mathcal {V}}_{k_2})$ and $Z^l({\mathcal {V}}^*_{k_2})$, where $l=G$ or *M*. Since a good latent space projection should facilitate distance-based classifiers, NNA is used to measure the goodness of the distance measure from the latent space projection $Z^l({\mathcal {V}}_{k_2})$ or $Z^l({\mathcal {V}}^*_{k_2})$. When the $\text {NNA}(Z^l({\mathcal {V}}_{k_2}))$ is greater than the $\text {NNA}(Z^l({\mathcal {V}}^*_{k_2}))$, the latent space projection $Z^l({\mathcal {V}}_{k_2})$ is more efficient for classification than the latent space projection $Z^l({\mathcal {V}}^*_{k_2})$ and vice versa. We use NMI to evaluate the consistency between the obtained clustering ${\hat{y}}^l({\mathcal {V}}_{k_2})$ or ${\hat{y}}^l({\mathcal {V}}^*_{k_2})$ and the true labels. A higher NMI indicates a better clustering result. In addition to using NNA in a supervised setting, we also adopted random forest to evaluate the classification performances of different methods. We calculate a random forest classifier’s average classification accuracy, denoted by RFA, under a 5-fold cross-validation framework.

Table 2 reports the number of critical genes $k_2$ after pruning, $k_2/k_1$, the ratio $k_2/p$, and the corresponding NNA, RFA, and NMI based on $Z^l({\mathcal {V}}_{k_2})$, $Z^l({\mathcal {V}}^*_{k_2})$ and $Z^{{M}}(X)$ for the four datasets. The results in Table  2 show that, in most cases, Strategy A only select a small percentage ($k_2/p$) of features, but achieve NNA, RFA, and NMI that are comparable to or even better than using all features. In view of $k_2/k_1$, Strategy A pruned over 25% features in ${\mathcal {V}}_{k_1}$ for the mECS, Kolod, and Usoskin datasets. In particular, for the Kolod dataset, Strategy A only requires 2 genes, accounting for $0.01\%$, to achieve the same performance based on all features. Also, for the Usoskin dataset, NNA, RFA, and NMI based on KC and Laplacian scores are higher than the benchmark.

**Table 2:** $k_2$, $k_2/k_1$, $k_2/p$, NNA, RFA and NMI based on $Z^l({\mathcal {V}}_{k_2})$, $Z^l({\mathcal {V}}^*_{k_2})$ and $Z^{\text {S}}(X)$ for the four data sets.

Data set	Latent space projection	$k_2$	$k_2/k_1$ (%)	$k_2/p$ (%)	NNA	RFA	NMI
mECS	$Z^S({\mathcal {V}}_{k_2})$	2860	50.49	31.82	0.97	0.96	0.84
($p=8989$)	$Z^S({\mathcal {V}}^*_{k_2})$	5595	69.89	62.24	0.95	0.96	0.85
	$Z^S(X)$				0.95	0.95	0.89
Kolod	$Z^G({\mathcal {V}}_{k_2})$	2	8	0.01	1.00	1.00	1.00
($p=13473$)	$Z^G({\mathcal {V}}^*_{k_2})$	10	28.57	0.07	1.00	1.00	1.00
	$Z^S(X)$				1.00	1.00	0.99
Pollen	$Z^G({\mathcal {V}}_{k_2})$	225	100	3.22	0.98	0.98	0.94
($p=6982$)	$Z^G({\mathcal {V}}^*_{k_2})$	115	100	1.65	0.98	0.98	0.91
	$Z^S(X)$				0.98	0.95	0.95
Usoskin	$Z^G({\mathcal {V}}_{k_2})$	65	41.94	0.37	0.99	0.99	0.96
($p=17772$)	$Z^G({\mathcal {V}}^*_{k_2})$	55	73.33	0.31	0.98	0.98	0.93
	$Z^S(X)$				0.94	0.96	0.74

**Figure 2:**
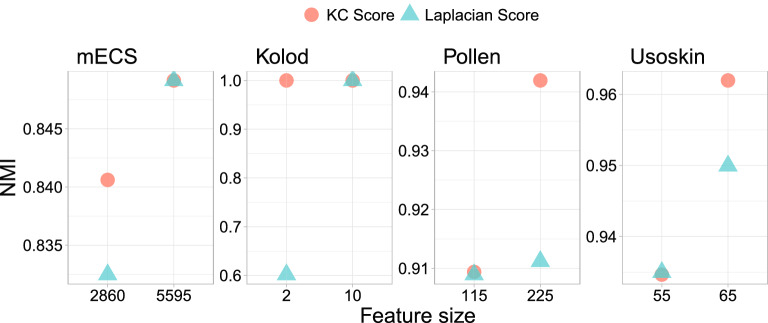
The NMIs based on the two $k_2$’s selected respectively by the KC and Laplacian Scores in Table 2.

To further evaluate the performance of KC and Laplacian Scores, we compare their NMIs based on the two $k_2$’s selected respectively by the KC and Laplacian Scores in Table 2, see Fig. 2. The results show that the KC Score has either comparable or better NMI for the four datasets than the Laplacian Score for both of the two $k_2$’s.

### Comparison of single and multi-resolution Gaussian kernels

**Figure 3:**
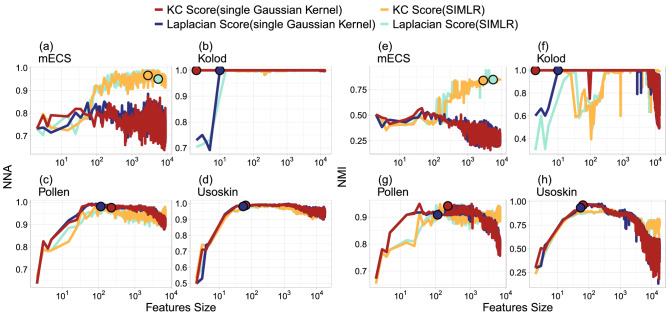
The NNA and NMI curves of Strategy A against different numbers(log scale) of genes based on the KC Score(single Gaussian kernel; SIMLR) versus Laplacian Score(single Gaussian kernel; SIMLR) for the four scRNA-seq datasets, where the circles in each subplot denote the locations of $k_2$.

Figure 3 illustrates the reason why the first step of Strategy A adopts the similarity matrix ($S^{G}$) estimated by the single Gaussian kernel method rather than the one ($S^{{M}}$) estimated by multi-resolution Gaussian kernels. Figure 3 presents the curves of NNA in (a)–(d) and NMI in (e)–(h) versus the feature size (log scale) for the four datasets. Each subfigure plots the curves of KC Score with single Gaussian kernel (red), KC Score with SIMLR (yellow), Laplacian Score with single Gaussian kernel (blue), and Laplacian Score with SIMLR (green). For the Kolod, Pollen, and Usoskin datasets, the KC Score and Laplacian Score with single Gaussian kernel perform better than the counterparties with SIMLR. The KC Score with single Gaussian kernel reaches the highest NNA and NMI much faster than the other three methods, especially for the Kolod. The corresponding highest NNA and NMI occur at $k^o=2$, where $k^o$ denotes the minimal *k* at which the NNA or NMI attains the highest peak of each method in Fig. 3. In contrast, the KC Score and Laplacian Score with SIMLR perform better than the single Gaussian kernel for the mECS dataset, and the highest NNA and NMI of KC Score with SIMLR were reached at ${k^o}>3000$, which are larger than the ${k^o}$ values of the other three methods. Hence, KC Score with single Gaussian kernel is recommended when the number of the critical genes is small, but KC Score with SIMLR is recommended when the number is large. One possible explanation of this phenomenon is that multi-resolution Gaussian kernels are designed for collecting a larger set of features than a single Gaussian kernel since different kernels might highlight various critical features. Nevertheless, if the number of helpful classification features is small, a single kernel might be good enough to identify these genes. This might explain why our numerical experiments reveal that KC score with a single Gaussian kernel performs better than the multi-kernel approach if $k^o$ is small. Moreover, each subfigure in Fig. 3 is also marked with the $k_2$ values of the KC Score and the Laplacian Score obtained from Table 2. The NMI and NNA at $k_2$ are higher than those at most of the other *k* values in each dataset, which indicates that the $k_2$ features recommended by Strategy A can produce satisfactory clustering performances for the four datasets.

## Discussion

This study proposes a KC Score to measure feature importance. The KC Score is designed by measuring the correlation between the original and the associated reconstruction genes expression based on the latent space obtained from SIMLR and t-SNE. A feature selection strategy is also developed for the KC Score or Laplacian Score to select the critical genes. The strategy is applied to four datasets. The results show that, when there are few critical genes, the latent space based on KC Score and single Gaussian kernel has the best performance. In contrast, the latent space based on SIMLR is recommended when the number of critical genes is relatively large.

**Figure 4:**
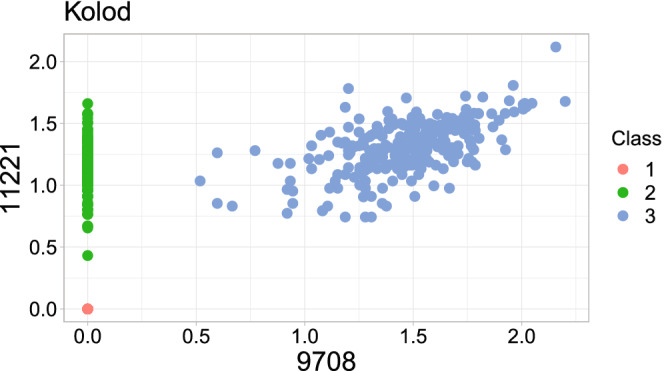
Scatter plot of the first two (the 9708th and 11221st) critical cell gene expressions for the Kolod dataset.

In particular, for the Kolod dataset, Strategy A with a KC Score only selects two critical genes to achieve perfect clustering, meaning that the corresponding NMI is 1. To gain more insights into how those two selected critical genes produce perfect clustering, Fig. 4 shows the scatter plot of the first two (the 9708th and 11221st) critical cell gene expressions. In Fig. 4, if both two cell genes’ expressions occur dropout, the cells are classified as Class 1 (red points); if only the 9708th cell gene expression is dropped, the cells are classified as Class 2 (green points); if neither of the two cell gene expressions dropout, the cells are classified as Class 3 (blue points). As the figure shows, the three classes are separated perfectly among the above three dropout patterns. This phenomenon shows that dropout patterns can provide helpful and informative signals for scRNA-seq clustering. In the literature, other studies also reported similar findings that dropout patterns might be a helpful signal in single-cell data analysis^8–10^. Nonetheless, dropout patterns may be less informative when they are very dispersed, as is common in other areas such as microbiome data.

**Figure 5:**
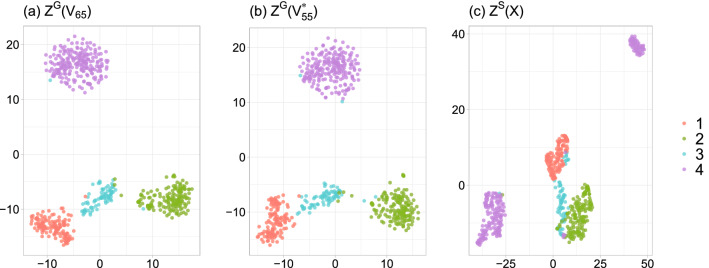
Three 2-dimensional latent spaces obtained by t-SNE for the Usoskin dataset: (**a**) $Z^G({\mathcal {V}}_{65})$, (**b**) $Z^G({\mathcal {V}}^*_{55})$, and (**c**) $Z^S(X)$.

Moreover, concerning the NMIs in Table 2 for the Usoskin dataset, the clustering performance improves markedly, from 0.74 to over 0.93, by proceeding feature selection. To visualize this finding, Fig. 5 shows the projections and clustering results in the three 2-dimensional latent spaces obtained by t-SNE: (a) $Z^G({\mathcal {V}}_{65})$, (b) $Z^G({\mathcal {V}}^*_{55})$, and (c) $Z^{{M}}(X)$. In Fig. 5, the clustering performances of $Z^G({\mathcal {V}}_{65})$ and $Z^G({\mathcal {V}}^*_{55})$ are shown to be comparable. However, compared to $Z^G({\mathcal {V}}_{65})$ and $Z^G({\mathcal {V}}^*_{55})$, the separability among the four classes in $Z^{{M}}(X)$ is relatively low, especially for the cells in Class 4 (purple points), since they are divided incorrectly into two groups. This finding highlights that identifying and using critical features for clustering is more effective than all features.

In the procedure of Strategy A, we adopted the unsupervised learning method SIMLR to cluster the cells. In SIMLR, one must conduct many matrix calculations with size $n\times n$, where *n* denotes the number of cells. Therefore, the computational cost is very expensive with a large *n*, which leads to a limitation of Strategy A. Table 3 presents the running hours spent in steps 1 and 2 of Strategy A when applying different methods to the four datasets. All the computations are conducted on servers with 2.40 GHz CPU, NVIDIA-SMI 430.50 GPU, and 126 GB RAM. One can see that the running hours of using SIMLR to compute the KC Score and Laplacian Score are roughly proportional to $n^2$. Hence, the running hours of the Kolod ($n=704$) and Usoskin ($n=622$) datasets dramatically increase compared to the other two datasets with smaller *n*. In addition, the running hours of SIMLR are remarkably more extensive than those of the associated single Gaussian kernel. Consequently, once the challenge of the heavy computational burden in SIMLR with a large *n* can be solved in the future, this limitation of Strategy A could be released.

**Table 3:** The running hours spent in steps 1 and 2 of Strategy A when applying different methods to the four datasets.

Dataset	*n*	KC Score (single Gaussian Kernel)	KC Score (SIMLR)	Laplacian Score (single Gaussian Kernel)	Laplacian Score (SIMLR)
mECS	182	0.09	5.90	0.09	5.93
Kolod	704	2.76	64.17	2.70	68.09
Pollen	249	0.12	7.21	0.12	7.33
Usoskin	622	3.39	76.02	3.46	77.43

**Figure 6:**
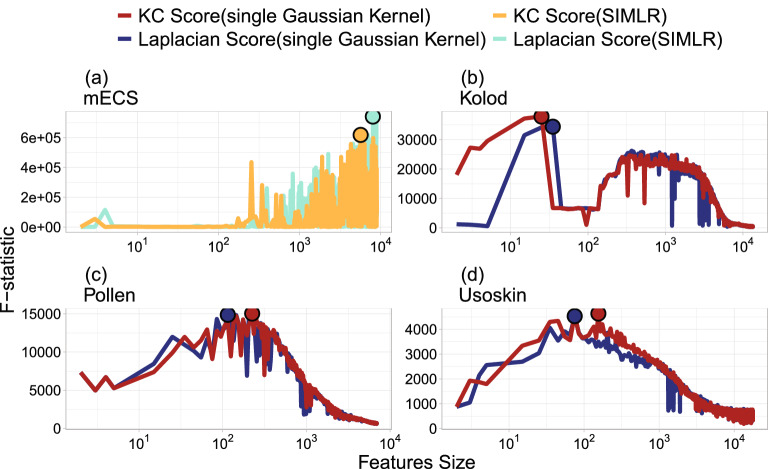
The MANOVA Pillai’s Trace statistic curves of Strategy A against different numbers (log scale) of genes based on the KC Score (single Gaussian kernel; SIMLR) versus Laplacian Score (single Gaussian kernel; SIMLR) for the four scRNA-seq datasets, where the circles denote the locations of the $k_1$’s for each method.

Figure 6 presents the MANOVA Pillai’s Trace statistic curves for the four datasets. From the figure, one can find that the ${\mathcal {F}}$ statistic is not a monotonic function of *k*. In particular, the curves tend to decrease as the number of features is large enough in the Kolod, Pollen, and Usoskin datasets. Moreover, it can be seen that the curves in Fig. 6 are not smooth around $k_1$ and severely fluctuate for $k\le k_1$ in the mECS, Pollen, and Usoskin datasets. Suppose we pruned genes via a similar method to step 3 of Strategy A, by replacing the role of NMI with the ${\mathcal {F}}$ statistic. In that case, we would need to develop a new test statistic to decide the critical value for selecting $k_2$. Doing so is beyond the scope of this study, and we leave it to our future work.

**Figure 7:**
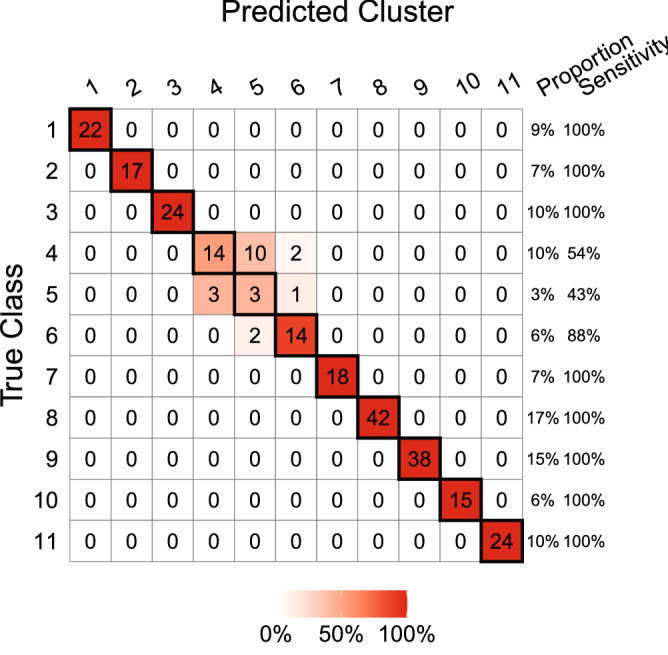
The confusion table of the clustering result of $Z^{G}({\mathcal {V}}_{k_2})$, where the clusters are rearranged with the highest accuracy.

In addition, we investigate the performance of strategy A in imbalanced data by delving into the most imbalanced one, Pollen, among the four datasets. The Pollen dataset consists of 11 classes, and one of the classes only contains 3% of cells in the dataset. Figure 7 shows the confusion table of the clustering result of $Z^{G}({\mathcal {V}}_{k_2})$ in Table 2, where the clusters are rearranged with the highest accuracy. The results in Fig. 7 reveal that the sensitivity of the 5th class is indeed affected by its low proportion of cells in the dataset. In general, the classification problem of imbalanced data is significantly challenging, even in supervised learning. In this study, since we considered an unsupervised learning task, the identification problem of a class with a small percentage of observations is even more difficult. Therefore, the low sensitivity for the class containing a low proportion of cells in the dataset revealed in Fig. 7 is not surprising. Further studies to improve the sensitivity of this scenario in supervised and unsupervised learning are needed in the future.

Note that the performance of Strategy A using KC Score highly relies on the classes being able to be separated in the projected latent space, because the KC Score is calculated by reconstruction from projections in the latent space. It is therefore necessary to overcome this limitation in order to extend the applicability of the proposed method. Also, step 3 of Strategy A is based on filtering out the most non-essential genes iteratively until a stopping criterion is met, so there is still room for further refinement in future studies.

## Methods

### Feature ranking criteria

#### Laplacian Score

The following notation is used for the data matrix ${\varvec{X}}$1where ${\varvec{x}}_{\varvec{.}k}=(x_{1k},x_{2k}, \dots , x_{nk})'$ denotes the *n* observations of the *k*th feature and ${\varvec{x}}_{i\varvec{.}}=(x_{i1},x_{i2}, \dots , x_{ip})$ denotes the *p* features of the *i*th observation. The Laplacian Score is a well-known unsupervised feature ranking method, which uses a similarity matrix^5^. The Laplacian Score of a given similarity matrix ${\varvec{S}}$ is defined as

2 $$\begin{aligned}&L_k =\frac{\tilde{{\varvec{x}}}_{\varvec{.}k}^T{\varvec{L}} \tilde{{\varvec{x}}}_{\varvec{.}k}}{\tilde{{\varvec{x}}}_{\varvec{.}k}^TD\tilde{{\varvec{x}}}_{\varvec{.}k}} \end{aligned}$$

where $D=\text {diag}({\varvec{S}}{\varvec{1}})$, ${\varvec{L}}={\varvec{D}}-{\varvec{S}}$, $\tilde{{\varvec{x}}}_{\varvec{.}k}={\varvec{x}}_{\varvec{.}k}-\frac{{\varvec{x}}_{\varvec{.}k}^TD{\varvec{1}}}{{\varvec{1}}^TD{\varvec{1}}}{\varvec{1}}$ and ${\varvec{1}}=(1,\dots ,1)'$. The smaller the Laplacian Score, the more important the feature is. The similarity matrix ${\varvec{S}}$ of the SIMLR method, denoted by $S^M(X)$, is constructed via the following optimization objective function and constraints:

3 $$\begin{aligned} \begin{aligned}{}&\text {min}_{{\varvec{S}},L,w}\sum _{i,j} D({\varvec{x}}_{i\varvec{.}},{\varvec{x}}_{j\varvec{.}})S_{ij}+\beta \Vert {\varvec{S}}\Vert _F^2+\gamma \mathbf{tr} (L^T(I_N-{\varvec{S}})L)+\rho \sum _l w_l \log {w_l}\\&\text {subject to } D({\varvec{x}}_{i\varvec{.}},{\varvec{x}}_{j\varvec{.}})=\sum _l w_l\Vert \phi _l({\varvec{x}}_{i\varvec{.}})-\phi _l({\varvec{x}}_{j\varvec{.}})\Vert _2^2,\ \sum _l w_l=1,\ w_l\ge 0,\\&L^TL=I_C,\ \sum _j S_{ij}=1,\ \text {and}\ S_{ij}\ge 0\ \forall (i,j) \end{aligned} \end{aligned}$$

where $\phi _l({\varvec{x}}_{i\varvec{.}})$ is the *l*-th kernel-induced implicit mapping of *i*th observation. We can rewrite $\Vert \phi _l({\varvec{x}}_{i\varvec{.}})-\phi _l({\varvec{x}}_{j\varvec{.}})\Vert _2^2$ as the representative of the kernel function

$$\begin{aligned} \Vert \phi _l({\varvec{x}}_{i\varvec{.}})-\phi _l({\varvec{x}}_{j\varvec{.}})\Vert _2^2&=\phi _l({\varvec{x}}_{i\varvec{.}})^T\phi _l({\varvec{x}}_{i\varvec{.}})+ \phi _l({\varvec{x}}_{j\varvec{.}})^T\phi _l({\varvec{x}}_{j\varvec{.}})-2 \phi _l({\varvec{x}}_{i\varvec{.}})^T\phi _l({\varvec{x}}_{j\varvec{.}})\\&=K_l^{\sigma }({\varvec{x}}_{i\varvec{.}},{\varvec{x}}_{i\varvec{.}})+K_l^{\sigma }({\varvec{x}}_{j\varvec{.}},{\varvec{x}}_{j\varvec{.}})-2K_l^{\sigma }({\varvec{x}}_{i\varvec{.}},{\varvec{x}}_{j\varvec{.}}), \end{aligned}$$

where the kernel functions are defined as

$$\begin{aligned} K_k^{\sigma }({\varvec{x}}_{i\varvec{.}},{\varvec{x}}_{j\varvec{.}})=\frac{1}{\sigma (\sigma _i+\sigma _j)\sqrt{2\pi }}\exp {\left( \frac{-\Vert {\varvec{x}}_{i\varvec{.}}-{\varvec{x}}_{j\varvec{.}}\Vert _2^2}{\sigma ^2(\sigma _i+\sigma _j)} \right) } \end{aligned}$$

with $\sigma _i= \sum _{j\in {KNN}_k(i)}\Vert {\varvec{x}}_{i\varvec{.}}-{\varvec{x}}_{j\varvec{.}}\Vert _2/k$ and ${KNN}_k(i)$ being the set of the top *k* neighbors of the *i*th observation. In practice, the parameters *k* and $\sigma$ in $K_k^{\sigma }(\cdot ,\cdot )$ are obtained from the combinations of $k\in \{10,12,14,\dots ,30\}$ and $\sigma \in \{1.0,1.25,\dots ,2.0\}$, which results in 55 different kernels.

#### Kriging-Correlation Score (KC Score)

The Kriging-Correlation Score (KC Score) proposed in this study aims to improve the efficiency of feature selection. We now introduce the algorithm for the KC score. Let ${\varvec{Z}}\in {\mathbb {R}}^{n\times d}$ be the *n* projections in a *d*-dimensional latent space of a dimension reduction method. Based on *Z*, we adopt the Automatic Fixed Rank Kriging (AutoFRK)^1^ to define the KC score. The details are as follows:

**Step 1**: Use ${\varvec{Z}}$ as the location inputs to generate the multi-resolution thin-plate spline basis matrix *G*. For each feature in data matrix ${\varvec{X}}$, fit the following spatial random effect model

$$\begin{aligned}&{\varvec{x}}_{\varvec{.}k} =G {\varvec{w}}_k+\varvec{\eta }_k \end{aligned}$$

where $G_{iw}=f_w(\varvec{z_{i\varvec{.}}})$, ${\varvec{w}}_k\sim N({\varvec{0}},M_k)$, and $\varvec{\eta }_k\sim N({\varvec{0}},\sigma _{\eta k}^2 I)$. In particular, $f_w(\cdot )$ denotes the multi-resolution thin-plate spline basis function and is defined as

$$\begin{aligned} f_w({\varvec{z}})=&{\left\{ \begin{array}{ll} 1; &{} w=1\\ {\varvec{z}}_{w} &{} w=2,\dots ,d+1\\ \lambda _{w-d-1}^{-1}\{\varvec{\phi }({\varvec{z}})-\varvec{\Phi {\tilde{Z}}({\tilde{Z}}'{\tilde{Z}}^{*})^{-1}{\tilde{z}}}'\}'{\varvec{v}}_{w-d-1} &{} w=d+2,\dots , n \end{array}\right. } \end{aligned}$$

where $$\tilde{{\varvec{Z}}}:=\begin{pmatrix}{\varvec{1}}&{\varvec{Z}}\end{pmatrix}, \tilde{{\varvec{z}}}:=(1,{\varvec{z}}), \varvec{\phi ({\varvec{z}})}:=(\phi _1(\varvec{{\varvec{z}}}),\dots ,\phi _n(\varvec{{\varvec{z}}}))', \varvec{\Phi }_{ik}=\phi _k{({\varvec{z}}_{i\varvec{.}})},$$ and

$$\begin{aligned} \phi _i({\varvec{z}})=&{\left\{ \begin{array}{ll} \frac{1}{12} \Vert {\varvec{z}}-{\varvec{z}}_{i\varvec{.}}\Vert ^3,&{} d=1\\ \frac{1}{8\pi } \Vert {\varvec{z}}-{\varvec{z}}_{i\varvec{.}}\Vert ^2\log {\Vert {\varvec{z}}-{\varvec{z}}_{i\varvec{.}}\Vert },&{} d=2\\ -\frac{1}{8} \Vert {\varvec{z}}-{\varvec{z}}_{i\varvec{.}}\Vert ,&{} d=3. \end{array}\right. } \end{aligned}$$

**Step 2**: The KC Score is defined as $({{\varvec{K}}}{{\varvec{C}}})_k = \widehat{\text {Cor}}({\varvec{x}}_{\varvec{.}k},\hat{{\varvec{x}}}_{\varvec{.}k}),$ where $\hat{{\varvec{x}}}_k$ are the fitted values obtained from Step 1.

### MANOVA Pillai’s Trace statistic

Let *G* be the number of classes and ${\hat{y}}_i\in \{1,\dots ,G\}$ be the clustering of observation *i*. The total of Sum of Cross-Products (SSCP) can be divided into ‘between’ and ‘within’ groups. That is,

$$\begin{aligned} \sum _{g=1}^G\sum _{\{i:{\hat{y}}_i=g\}}({\varvec{z}}_{i \varvec{.}}-\bar{{\varvec{z}}})'({\varvec{z}}_{i \varvec{.}}-\bar{{\varvec{z}}})&= \sum _{g=1}^G\sum _{\{i:{\hat{y}}_i=g\}} (\bar{{\varvec{z}}}^{(g)}-\bar{{\varvec{z}}})'(\bar{{\varvec{z}}}^{(g)}-\bar{{\varvec{z}}})+ \sum _{g=1}^G\sum _{\{i:{\hat{y}}_i=g\}} ({\varvec{z}}_{i \varvec{.}}-\bar{{\varvec{z}}}^{(g)})'({\varvec{z}}_{i \varvec{.}}-\bar{{\varvec{z}}}^{(g)})\\&=\text {Between Groups SSCP}+\text {Within Groups SSCP}\\&=B+W \end{aligned}$$

where $\bar{{\varvec{z}}}=\sum _{i=1}^n {\varvec{z}}_{i \varvec{.}}/n$ and $\bar{{\varvec{z}}}^{(g)}=\sum _{\{i:{\hat{y}}_i=g\}} {\varvec{z}}_{i \varvec{.}}/|\{i:{\hat{y}}_i=g\}|$. The Pillai’s Trace statistic is defined as ${\mathcal {F}}=\text {trace}(B(B+W)^{-1}).$

### Evaluation criteria

1. NNA
  We consider a performance metric in a supervised setting, namely the Nearest Neighbor Accuracy (NNA). We use 5-fold cross-validation on the transformed matrix $Z^l({\mathcal {V}}_j)$ and its true labels *y*, where $l=G$ or *M*. For each trial, we use four folders as the training set and the remaining one as the validation set. For each cell in the validation set, its class is assigned as the label of the training set object that is smallest Euclidean distance from the target cell. The $\text {NNA}^l_j$ is defined as the average accuracy of the five validation sets.
2. NMI
  Normalized Mutual Information (NMI) is a measure to evaluate the clustering consistency between the two clusters $U=(U_1,\dots ,U_n)$ and $V=(V_1,\dots ,V_n)$. Let *P* and *Q* denote the number of labels in *U* and *V*, respectively. Let $n_{pq}=|\{i=1,\dots ,n:\ U_i=p,\ V_i=q\}|$, $n_{p+}=\sum _{q=1}^Qn_{pq}$, and $n_{+q}=\sum _{p=1}^Pn_{pq}$. The NMI is defined as
  $$\begin{aligned} \text {NMI}(U,V)=\frac{I(U,V)}{\max {\left( H(U),H(V)\right) }}, \end{aligned}$$
  where
  $$\begin{aligned} I(U,V)&=\sum _{p=1}^{P} \sum _{q=1}^{Q} \frac{n_{pq}}{n}\log {\frac{n_{pq}/n}{n_{p+}n_{+q}/n^2}},\ \ H(U)~=~-\sum _{p=1}^P \frac{n_{p+}}{n}\log {\frac{n_{p+}}{n}}, \ \mathrm{and}\ \ H(V)~=~-\sum _{q=1}^Q \frac{n_{+q}}{n}\log {\frac{n_{+q}}{n}}. \end{aligned}$$
